# Extrapolation of Adult Evidence to Pediatric Practice: Bridging the Gap in Pediatric Heart Failure Pharmacotherapy

**DOI:** 10.3390/children13091223

**Published:** 2026-09-10

**Authors:** Adelina-Mihaela Sorescu, Cristina Isabel Viorica Ghiță, Smaranda Stoleru, Gabriela Duică, Alin Marcel Nicolescu, Eliza Elena Cinteză, Ion Fulga, Oana Andreia Coman

**Affiliations:** 1 Department I—Functional Sciences, Pharmacology, Clinical Pharmacology and Pharmacotherapy, “Carol Davila” University of Medicine and Pharmacy, 020021 Bucharest, Romania; adelina-mihaela.enache@drd.umfcd.ro (A.-M.S.); smaranda.stoleru@umfcd.ro (S.S.); ion.fulga@umfcd.ro (I.F.); oana.coman@umfcd.ro (O.A.C.); 2 Pediatrics Department, “Carol Davila” University of Medicine and Pharmacy, 020021 Bucharest, Romania; gabriela.duica@umfcd.ro (G.D.); eliza.cinteza@umfcd.ro (E.E.C.); 3 “Marie S. Curie” Clinical Emergency Hospital for Children, 041451 Bucharest, Romania; nicolescu_a@yahoo.com

**Keywords:** heart failure, adults, children, evidence gap, future directions

## Abstract

**Highlights:**

**What are the main findings?**

• Although contemporary pharmacological management targets similar neurohormonal pathways in adult, as well as pediatric patients, the evidence supporting these therapies differs substantially in the two populations.
• While adult heart failure treatment is supported by numerous large randomized clinical trials and well-established GDMT, pediatric recommendations continue to rely largely on extrapolation from adult studies, observational evidence and expert consensus.
• The marked heterogeneity of pediatric heart failure, together with developmental differences in myocardial physiology, pharmacokinetics and pharmacodynamics, highlights the limitations of directly translating adult evidence into pediatric clinical practice.

**What is the implication of the main finding?**

• Generating robust evidence (well-designed, multicenter pediatric clinical trials) is essential to optimize pharmacological therapy and ultimately improve outcomes for children with heart failure.

**Abstract:**

Heart failure (HF) is a complex clinical syndrome that represents an increasingly relevant health issue, associated with substantial morbidity and mortality in pediatric patients. While considerable therapeutic advances have transformed the prognosis of adult patients with heart failure over the past three decades, progress in pediatric heart failure has been considerably slower. In adult patients, large randomized controlled trials (RCTs) have established angiotensin-converting enzyme inhibitors (ACE inhibitors), beta-blockers, mineralocorticoid receptor antagonists (MRAs), angiotensin receptor-neprilysin inhibitors (ARNIs), and, more recently, sodium-glucose cotransporter-2 (SGLT2) inhibitors as the cornerstone of guideline-directed medical therapy. Evidence supporting pharmacological therapy in pediatric heart failure remains scarce and frequently inconclusive, with most available studies being limited by small sample sizes and heterogeneous patient populations. Therefore, heart failure treatment in children is mostly extrapolated from adult studies and guidelines. However, developmental differences in myocardial structure and function, neurohormonal signaling, pharmacokinetics and pharmacodynamics may substantially influence therapeutic response. In addition, the rarity and the etiological heterogeneity of pediatric HF create major methodological challenges for adequately powered clinical trials. This review compares the pharmacological management of HF in adult and pediatric populations, examines the strength of evidence for contemporary therapies and discusses the biological and methodological limitations of translating adult evidence into pediatric practice. Multicenter collaborative studies and well-designed pediatric randomized controlled trials are essential for establishing truly evidence-based pharmacological strategies for children with HF.

## 1. Introduction

Heart failure (HF) in children has been considered an increasingly relevant health issue, with rising mortality [1,2]. While considerable therapeutic advances have transformed the prognosis of adult patients with heart failure over the past three decades, progress in pediatric heart failure has been considerably slower. This discrepancy is largely attributable to the rarity and heterogeneity of pediatric heart failure, which have limited the development of robust clinical evidence and evidence-based treatment recommendations.

Several definitions of HF have been proposed. The 2021 European Society of Cardiology guideline defines HF as a syndrome (with specific signs and symptoms) associated with several structural and/or functional cardiac anomalies, resulting in “elevated intracardiac pressures and/or inadequate cardiac output at rest and/or during exercise” [3]. Due to the heterogeneity of the disease, the age at onset, clinical and diagnostic characteristics, as well as the treatment may vary [4].

Epidemiological studies have reported an incidence of HF of up to 7.4 cases per 100,000 children in some countries, such as Taiwan [5]. In adults, HF most commonly results from ischemic heart disease, hypertension, valvular disease, and diabetes mellitus. Pediatric HF, however, encompasses a heterogeneous spectrum of disorders, including congenital heart disease [6], cardiomyopathies, acute myocarditis, inherited metabolic disorders, and genetic syndromes, with the relative contribution of each etiology varying according to the age at diagnosis [7,8,9,10,11,12,13,14].

The diagnosis of HF can be challenging, as the presentation can be highly nonspecific and age-related, ranging from mild respiratory or gastrointestinal symptoms to severe cardiogenic shock, depending on age, disease progression, and etiology [15,16,17,18]. While in infants, the key issues are respiratory symptoms, feeding difficulties, and poor weight gain, in older children, the clinical presentation more closely resembles that observed in adult patients [7,19,20].

During the last three decades, the management of adult heart failure has undergone a remarkable evolution. Large randomized controlled trials (RCTs) have established angiotensin-converting enzyme inhibitors (ACE inhibitors), beta-blockers, mineralocorticoid receptor antagonists (MRAs), angiotensin receptor-neprilysin inhibitors (ARNIs), and, more recently, sodium-glucose cotransporter-2 (SGLT2) inhibitors as the cornerstone of guideline-directed medical therapy. These therapies have consistently demonstrated a decrease in mortality, hospitalization rates, and symptom burden, fundamentally changing the natural history of heart failure with reduced ejection fraction.

Evidence supporting pharmacological therapy in pediatric heart failure remains scarce and frequently inconclusive, with most available studies being limited by small sample sizes and heterogeneous patient populations [21]. Over the years, differences between adult and pediatric patients have been highlighted regarding the etiology of dilated cardiomyopathy, pathophysiological and molecular processes, and drug metabolism and response to medication in children [1,15].

When it comes to the treatment of heart failure in children, few studies have been published, and current recommendations are largely based on expert consensus rather than extensive clinical studies. Because of this substantial lack of evidence, adult treatments and guideline recommendations [3,22] are often applied to pediatric patients [23]. Therefore, current pediatric management relies largely on expert consensus and off-label drug use, despite increasing recognition that developmental differences may significantly influence both drug efficacy and safety.

Pediatric heart failure treatment has remained relatively unchanged over the years; the main drug therapies still include ACE inhibitors, diuretics, MRAs and beta-blockers, either alone or in association. Apart from these drug classes, inotropes are used in severe or decompensated heart failure [3,19,24,25]. Etiology-specific treatments, including antioxidants such as Coenzyme Q10 and carnitine supplements, have also been investigated in patients with dilated cardiomyopathy, although the supporting pediatric evidence remains limited [8,26,27,28,29]. These therapies are supported by substantially stronger evidence in adults, whereas pediatric use is based on limited data, expert consensus and extrapolation from adult studies [30].

The absence of robust pediatric clinical trials represents one of the greatest challenges in pediatric cardiology. Small patient populations, disease heterogeneity, ethical considerations, and difficulties in trial design have all contributed to the scarcity of randomized controlled studies. As a result, important questions remain regarding the optimal pharmacological management of children with heart failure and the applicability of adult evidence to pediatric populations.

This narrative review was conducted to summarize and compare current evidence regarding the pharmacological management of heart failure in adult and pediatric populations. A structured literature search was performed using PubMed/MEDLINE and Scopus databases. The search covered publications from January 1999 to March 2026 and focused on the pharmacological treatment of heart failure in adult and pediatric patients. Search terms included combinations of “heart failure”, “pediatric heart failure”, “dilated cardiomyopathy”, “pediatric dilated cardiomyopathy”, “treatment”, “ACE inhibitors”, “beta-blockers”, “mineralocorticoid receptor antagonists”, “sacubitril/valsartan”, “SGLT2 inhibitors”, “ivabradine” and “diuretics”, combined using the Boolean operators AND and OR as appropriate.

Original clinical studies, randomized controlled trials, prospective and retrospective observational studies, systematic reviews, narrative reviews, clinical guidelines and scientific statements relevant to the pharmacological management of adult or pediatric heart failure were considered. Whenever original studies were available, they were preferentially used to assess treatment efficacy, safety and clinical outcomes, whereas systematic and narrative reviews were primarily used to provide contextual information and to identify additional relevant primary studies. Only articles published in English were included. A small number of potentially relevant publications could not be assessed because the full text was not available through open-access sources or institutional subscriptions. Emphasis was placed on studies providing pediatric-specific data on efficacy, safety, pharmacokinetics, pharmacodynamics, or clinical outcomes. The selected studies were also reviewed for relevant data on clinical characteristics and pathophysiology. Studies unrelated to heart failure pharmacotherapy or without relevance to the adult-to-pediatric evidence translation addressed in this review were excluded. Original studies were preferentially used to assess treatment efficacy, safety and clinical outcomes. Review articles were primarily used to provide background and contextual information and to identify additional relevant primary studies. Whenever original evidence was available, information regarding treatment effects was derived from the primary publications rather than from secondary sources.

Following the keyword search, articles were selected based on their relevance to the objectives of the review. Titles and abstracts were screened for relevance to the objectives of this review, followed by full-text assessment of potentially relevant publications. Reference lists of key articles, guidelines and reviews were additionally screened to identify relevant studies not captured in the initial database search.

This study was designed as a narrative review, not as a systematic review or meta-analysis. No formal risk-of-bias assessment was performed due to the heterogeneity of the studies available in the literature [31]. Artificial intelligence (AI) (ChatGPT, OpenAI, GPT-5.5) was used exclusively to assist in the design and drafting of schematic figures. The contents of the figures were reviewed, edited and validated after generation by the AI tool to ensure scientific accuracy and consistency with the cited literature.

**Figure 1:**
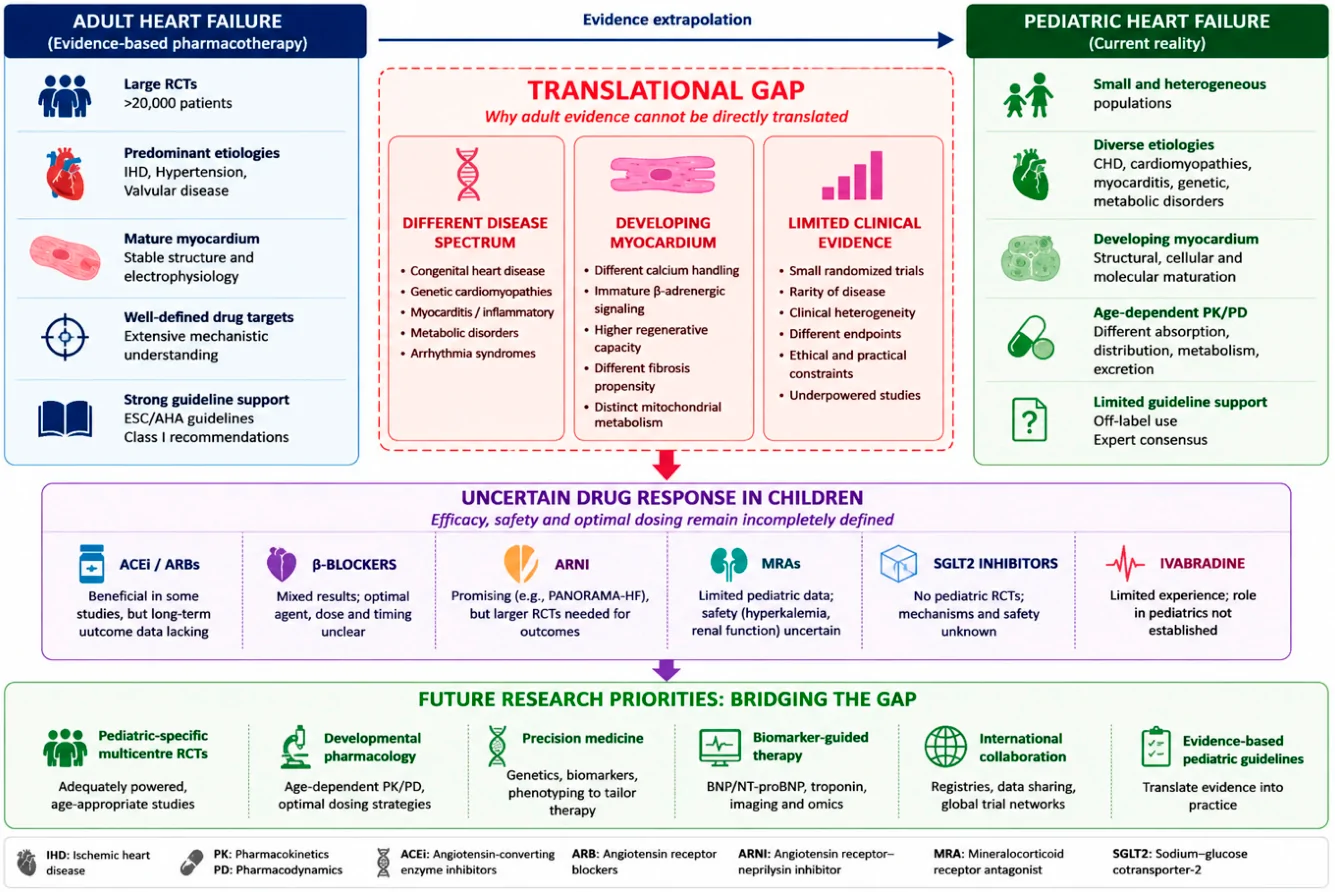
Summary of the main evidence gaps regarding pharmacological treatment in pediatric heart failure.

This review aims to critically compare the pharmacological management of heart failure in adults and children, summarize the current evidence supporting contemporary medical therapies, discuss the limitations of extrapolating adult data to pediatric patients, and highlight the urgent need for well-designed multicenter randomized clinical trials to establish evidence-based treatment strategies for pediatric heart failure (Figure 1).

## 2. Heart Failure: One Clinical Syndrome, Different Diseases

Given the remarkable etiological heterogeneity of heart failure across different age groups (Table 1), establishing the underlying etiology of heart failure is a fundamental step in the diagnostic evaluation and subsequent management of both adult and pediatric patients. Identifying the cause of heart failure is particularly important because some etiologies, such as certain congenital structural abnormalities, are potentially reversible through corrective interventions, whereas others are characterized by progressive myocardial dysfunction despite optimal medical therapy [32]. Consequently, etiological diagnosis plays an important role in guiding therapeutic strategies and determining both short- and long-term prognosis.

### 2.1. Heart Failure in Adults

The etiology of heart failure differs substantially between adult and pediatric populations (Table 2). In adult patients, heart failure is broadly classified as ischemic and non-ischemic [33]. Ischemic heart disease, particularly coronary artery disease and acute myocardial infarction, remains the leading cause of heart failure worldwide. Non-ischemic causes include hypertension, tachyarrhythmias, valvular disease and a wide range of systemic conditions, such as severe infections, metabolic disorders and chronic respiratory disease, all of which may contribute to myocardial dysfunction and development of heart failure in adult patients [3,15,33].

### 2.2. Heart Failure in Children

In contrast, pediatric heart failure is characterized by a considerably greater etiological heterogeneity. Congenital heart disease (CHD) represents the main underlying cause, accounting for more than half of the hospital admissions for heart failure in pediatric cardiology [15,19,34]. Beyond CHD, pediatric heart failure may result from a diverse spectrum of disorders, including primary and secondary cardiomyopathies, inherited metabolic and genetic diseases, myocarditis, rhythm and conduction abnormalities, as well as systemic inflammatory or infectious conditions [19,35,36]. This broad etiological diversity contributes to substantial variability in clinical presentation, disease progression and therapeutic response, distinguishing pediatric heart failure from adult heart failure [6,35,37].

The etiology of pediatric heart failure also varies considerably according to age. Congenital heart disease is the predominant cause of heart failure in neonates and infants. In children aged 2–5 years, cardiomyopathies, myocarditis, tachyarrhythmias and Kawasaki disease become increasingly prevalent, while in older children, cardiomyopathies remain the leading cause of heart failure, followed by rhythm and conduction abnormalities and systemic diseases [6].

Consequently, pediatric heart failure should not be regarded as a single disease, but rather as a spectrum of disorders with distinct pathophysiological mechanisms, clinical presentations and therapeutic approaches [3].

### 2.3. Why These Differences Matter

Although adult and pediatric heart failure arise from markedly different etiologies, both ultimately converge on a common final pathway, characterized by ventricular dysfunction, neurohormonal activation and progressive cardiac remodeling [17,38,39]. However, important developmental differences in myocardial biology and compensatory mechanisms suggest that these shared pathways may not be activated or modulated identically across age groups. Understanding these similarities and differences is essential for interpreting the efficacy of pharmacological therapies in pediatric heart failure [40].

## 3. Pathophysiology of Heart Failure

### 3.1. Common Pathophysiological Mechanisms

Cardiac physiology undergoes profound changes throughout fetal life, infancy, childhood and adolescence. Consequently, both normal cardiac function and the pathophysiological mechanisms that contribute to heart failure vary according to the stage of myocardial development. Together with the marked etiological heterogeneity of pediatric heart failure, these developmental differences contribute to complex and dynamic pathophysiological processes [41].

Heart failure is characterized by the inability of the heart to generate sufficient cardiac output to meet the metabolic demands of the body or to do so at the expense of elevated ventricular filling pressures. Although the underlying causes differ substantially between adults and children, the progression of heart failure is driven by several common pathophysiological mechanisms.

In a comprehensive review, Knudson and Cabrera identified four main mechanisms contributing to the development of pediatric heart failure: systolic dysfunction, characterized by impaired ventricular contractility; diastolic dysfunction, resulting from abnormal ventricular relaxation and reduced compliance; pulmonary volume overload, most commonly associated with congenital heart defects involving left-to-right shunts; and intra- or extracardiac mixing of oxygenated and deoxygenated blood, typically observed in complex congenital heart disease [42].

**Figure 2:**
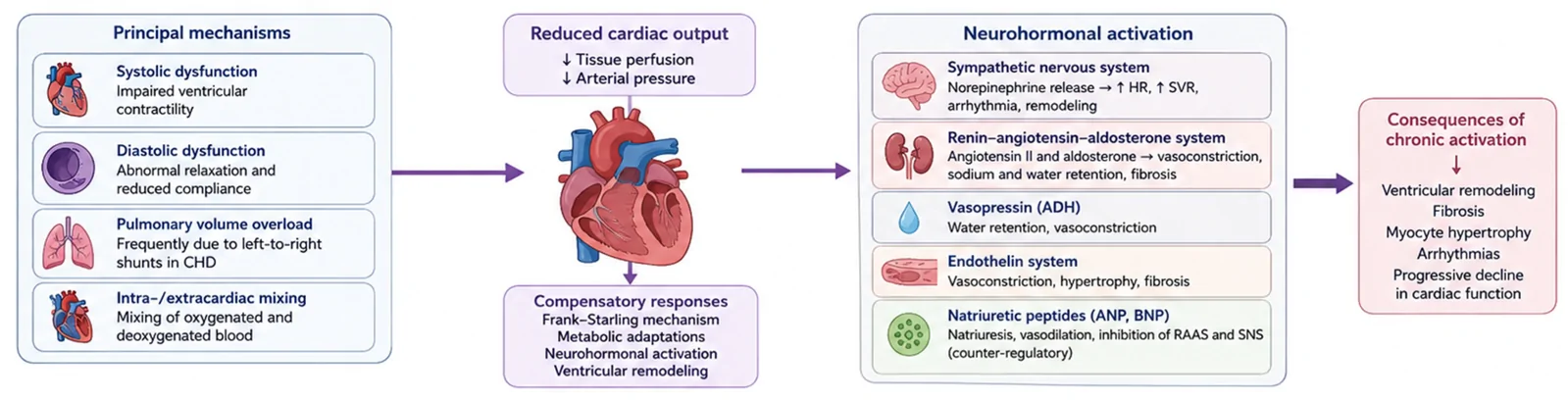
Pathophysiology and neurohormonal activation in adult vs. pediatric heart failure.

Regardless of the initiating mechanism, impaired cardiac performance results in reduced tissue perfusion. Consequently, a series of compensatory responses are activated to maintain systemic blood pressure and organ perfusion. These adaptive mechanisms include the Frank-Starling mechanism, metabolic adaptations, activation of neurohormonal pathways and ventricular remodeling [38,43]. While these responses initially preserve systemic perfusion, persistent activation ultimately becomes maladaptive, promoting progressive ventricular remodeling, myocardial fibrosis and dysfunction (Figure 2).

### 3.2. Developmental Differences Between Adult and Pediatric Heart Failure

Although adult and pediatric heart failure share the same neurohormonal pathways, the biological substrate differs considerably [40]. Unlike the mature myocardium, the pediatric heart undergoes continuous structural, cellular and molecular adaptations throughout infancy and childhood. Consequently, myocardial adaptation to injury, ventricular remodeling and the response to neurohormonal activation are influenced not only by the underlying disease, but also by the developmental stage.

Understanding the differences between adult and pediatric heart failure has important therapeutic implications [40,41]. Etiology represents one of the major differences between adult and pediatric heart failure, as different initiating mechanisms can lead to different patterns of myocardial injury [32,40]. In addition, the pediatric myocardium continues to undergo developmental processes, as the immature myocardium has distinct structural, metabolic and developmental characteristics, making the pathophysiology of heart failure in children fundamentally different from that of the adult heart [34,44]. At a molecular level, the pediatric myocardium differs in sarcomeric protein composition, calcium handling, mitochondrial metabolism and beta-adrenergic signaling, resulting in distinct patterns of cardiac remodeling and drug responsiveness [34,45].

**Figure 3:**
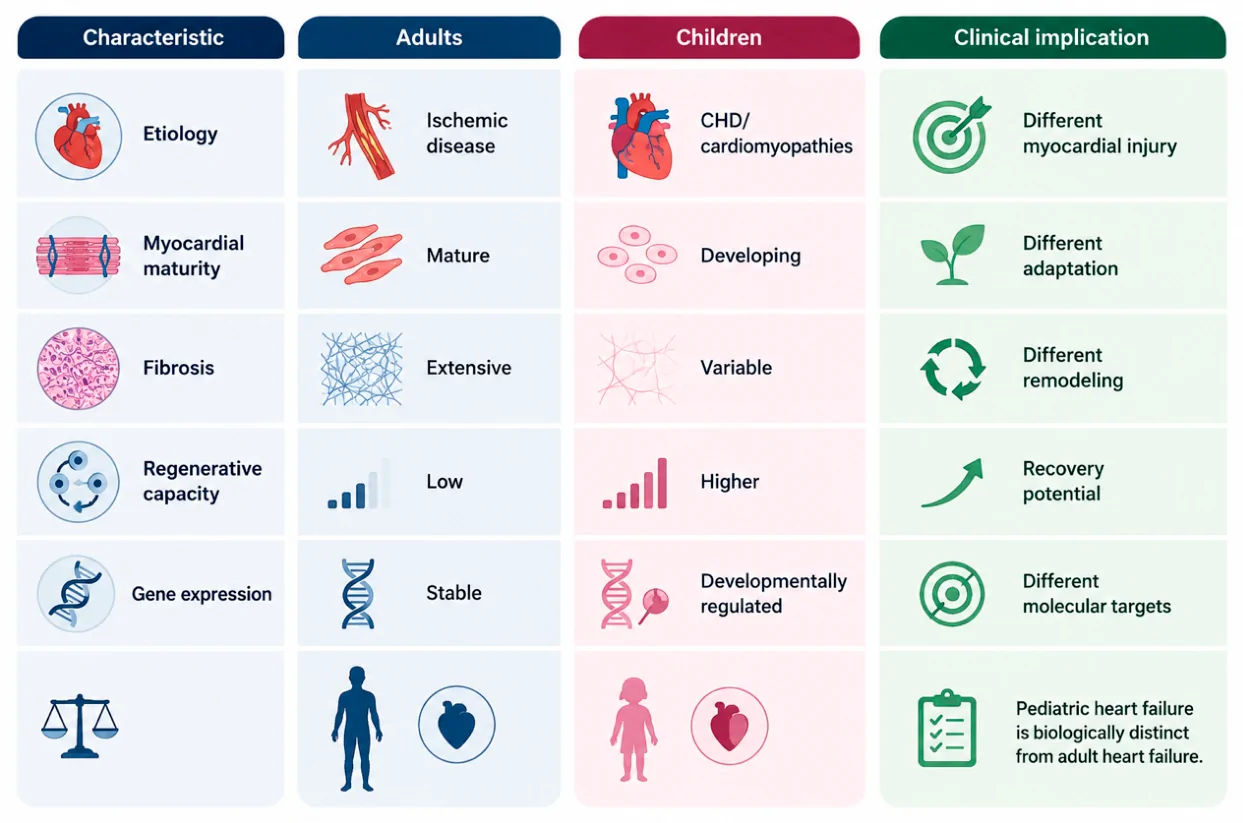
Developmental differences between adult and pediatric heart failure.

Although important differences in myocardial remodeling, fibrosis, regenerative capacity and gene expression have been identified, many aspects of pediatric heart failure biology remain poorly understood [46]. Consequently, further translational and clinical studies are required to clarify these developmental mechanisms and their involvement in pharmacological therapy. Figure 3 summarizes the main developmental differences between adult and pediatric heart failure.

### 3.3. Implications for Pharmacological Therapy

The developmental and molecular differences between adult and pediatric heart failure have important implications for pharmacological therapy. Although current pediatric treatment strategies largely target the same neurohormonal pathways as those seen in adult patients, the distinct biology of the developing myocardium may influence drug efficacy, safety and long-term outcomes [46]. While it may be reasonable to extrapolate pharmacological therapies from adult guidelines and practices, given the molecular and pathophysiological differences between adult and pediatric patients, it is difficult to determine their true efficacy and safety profiles in children [36]. In addition to developmental differences in myocardial biology, age-dependent changes in drug absorption, distribution and metabolism further contribute to the variability in pharmacological response [32,36,46].

Despite these limitations, pharmacological management of pediatric heart failure continues to rely largely on agents originally developed and validated in adult populations. Understanding the mechanisms of action, supporting evidence and current recommendations for these therapies is therefore essential to appreciate both their benefits and their limitations in pediatric practice [1,17].

## 4. Clinical Presentation

The clinical presentation of heart failure in children is highly variable and strongly influenced by age. In older children and adolescents, heart failure may present with exercise intolerance, fatigue, chest discomfort, respiratory symptoms, or signs of systemic congestion, including peripheral edema and hepatomegaly [30]. In contrast, neonates and infants frequently exhibit less specific manifestations, such as irritability, poor general appearance, feeding difficulties, prolonged feeding duration, and excessive sweating during feeding. These symptoms may subsequently result in inadequate caloric intake and failure to thrive [32,47]. The considerable age-related variability and nonspecific nature of these manifestations may complicate the early recognition of pediatric heart failure and contribute to delayed diagnosis or underdiagnosis [16,17].

## 5. Pharmacological Treatment of Heart Failure in Adults vs. Children

### 5.1. Principles of Therapy

The management of heart failure is inherently complex, owing to the wide spectrum of underlying etiologies and the intricate pathophysiological mechanisms involved in disease development and progression [48]. The primary goals of treatment are to address the underlying cause whenever possible, alleviate symptoms, improve functional status and quality of life, slow disease progression and ultimately improve survival [16,19]. However, as many causes of heart failure are irreversible, etiological treatment is often not feasible.

Consequently, contemporary pharmacological management is primarily based on targeting the maladaptive pathophysiological processes. Current therapies focus predominantly on modulating neurohormonal activation, while optimizing cardiac performance and limiting adverse ventricular remodeling [48,49].

The main therapeutic objectives include reducing preload and afterload, improving ventricular filling and cardiac output, optimizing myocardial oxygen consumption, controlling heart rate and rhythm and preventing progressive ventricular remodeling. Collectively, these interventions aim not only to improve symptoms, but also to mitigate the adverse effects of neurohormonal activation and progressive myocardial dysfunction that characterizes chronic heart failure [48].

Unlike adult heart failure, where treatment recommendations are supported by numerous large randomized controlled trials, pharmacological management in children remains largely based on extrapolation from adult practices [6]. Current international statements for pediatric heart failure rely predominantly on adult data, complemented by small observational pediatric studies and expert consensus. Only a few RCTs have been conducted on pediatric heart failure (Table 3). Although this strategy is often necessary in clinical practice, it highlights the urgent need for high-quality pediatric clinical trials to establish evidence-based therapeutic recommendations specifically for children with heart failure.

The main drug classes include ACE inhibitors and angiotensin receptor blockers (ARBs), beta-blockers, MRAs, ARNIs, sodium-glucose cotransporter-2 inhibitors (SGLT2 inhibitors) and diuretics. The evidence supporting each of these therapies in adults and children is discussed below.

### 5.2. ACE Inhibitors/MRAs/ARNI

The beneficial effects of angiotensin-converting enzyme (ACE) inhibitors are primarily mediated by reduced formation of angiotensin II and the consequent decrease in aldosterone secretion. By attenuating angiotensin II-mediated vasoconstriction, ACE inhibitors reduce preload through venodilation and decrease afterload through peripheral arterial vasodilation. In addition to these hemodynamic effects, suppression of the renin–angiotensin–aldosterone system limits myocardial fibrosis and adverse ventricular remodeling, thereby contributing to the long-term cardioprotective effects of ACE inhibitor therapy [53].

In adult patients, ACE inhibitors represent the cornerstone of guideline-directed medical therapy (GDMT), as they were the first drug class with proven efficacy in this disease [3]. Accordingly, current guidelines present ACE inhibitors as a class I, Level A recommendation. Similarly, MRAs also carry a class I, Level A recommendation supported by numerous RCTs [3]. In the same guidelines, ARNI therapy represents a class I, Level B recommendation, despite favorable results reported across several clinical studies. The landmark PARADIGM-HF trial demonstrated that sacubitril/valsartan was superior to enalapril in reducing cardiovascular mortality and heart failure hospitalizations, while also showing a good safety profile.

In pediatric heart failure, ACE inhibitors are among the most frequently prescribed medications, being used in approximately 70% of the affected children [24,25,54]. Among the available agents, captopril and enalapril remain the most used ACE inhibitors in clinical practice, due to their extensive clinical experience and favorable safety profile [53].

Although the evidence supporting ACE inhibitor therapy in children is considerably less robust than in adult patients, numerous observational studies and smaller clinical investigations have reported favorable effects across a wide range of heart failure etiologies [6,30]. In a prospective study, Mori et al. demonstrated that ACE inhibitor therapy improved left ventricular dimensions in children with volume-overload lesions, suggesting a beneficial effect on ventricular remodeling [55].

No randomized controlled trials have evaluated the efficacy of ACE inhibitors in pediatric heart failure. Despite the absence of high-powered pediatric randomized controlled trials, ACE inhibitors are widely used in pediatric heart failure, largely based on observational evidence, clinical experience, expert consensus and extrapolation from adult heart failure studies [32,36].

By blocking mineralocorticoid receptors, mineralocorticoid receptor antagonists (MRAs) counteract the effects of aldosterone, thereby reducing sodium and water retention, intravascular volume, and cardiac preload [56].

Evidence supporting the use of MRAs in pediatric heart failure remains limited [17,19]. Limited pediatric observational data suggest potential symptomatic benefit and acceptable tolerability [23]. Consequently, current pediatric practice largely reflects extrapolation from the robust evidence established in adult populations. This represents another important example of the evidence gap that continues to characterize pharmacological management in pediatric heart failure.

Sacubitril/valsartan is an angiotensin receptor–neprilysin inhibitor (ARNI) combining sacubitril, a neprilysin inhibitor, with valsartan, an angiotensin II receptor blocker. Neprilysin inhibition increases the availability of endogenous natriuretic peptides, thereby promoting natriuresis and vasodilation, whereas blockade of angiotensin II receptors counteracts the maladaptive effects of renin–angiotensin–aldosterone system activation. Together, these complementary mechanisms reduce cardiac loading conditions and limit adverse ventricular remodeling [52].

The efficacy and safety of sacubitril/valsartan in pediatric patients were evaluated in the PANORAMA-HF trial, a multicenter, randomized, double-blind clinical trial comparing sacubitril/valsartan with enalapril. Although treatment with sacubitril/valsartan resulted in significant clinical improvement, it did not demonstrate statistically significant superiority over enalapril for the primary efficacy endpoint [52]. These findings support the feasibility and tolerability of sacubitril/valsartan in pediatric heart failure and suggest potential clinical benefit. However, PANORAMA-HF did not demonstrate superiority over enalapril for the primary efficacy endpoint [57]. Additional adequately powered randomized clinical trials are required to establish its superiority over conventional ACE inhibitor therapy and better define the role of ARNIs in routine pediatric practice.

### 5.3. Beta-Blockers

Beta-blockers are a cornerstone of GDMT for adults with heart failure with reduced ejection fraction, in which they reduce mortality, hospitalizations and attenuate ventricular remodeling. The therapeutic role of beta-blockers in heart failure is primarily based on counteracting the deleterious effects of chronic sympathetic nervous system activation. By reducing excessive adrenergic stimulation, beta-blockers decrease heart rate and myocardial oxygen demand, limit further myocardial injury, and attenuate adverse ventricular remodeling [58].

In contrast, the role of beta-blockers in pediatric heart failure remains considerably less well defined. Although widely prescribed in adults, their use in children has historically been much less frequent, with early reports indicating that only approximately 5% of pediatric patients received beta-blocker therapy [24,32,59]. This difference is thought to reflect the distinct etiological spectrum of pediatric heart failure, which is predominantly non-ischemic, in contrast to the ischemic etiology that characterizes most adult heart failure. Therefore, the benefits observed in adults cannot be assumed to apply to pediatric patients [32,36].

Clinical studies evaluating beta-blockers in pediatric heart failure have produced conflicting results. While several observational studies and smaller clinical investigations have reported improvements in symptoms, ventricular function, and clinical outcomes [25,49], the overall evidence remains limited. A Cochrane systematic review concluded that the available data were insufficient to establish definitive recommendations, although observational studies suggest potential benefit, whereas randomized pediatric evidence remains inconclusive [60].

The largest randomized, double-blind, placebo-controlled trial evaluating carvedilol in children with heart failure, conducted by Shaddy et al., failed to demonstrate a significant advantage over placebo, while showing an acceptable safety profile [50]. The authors suggested that the heterogeneous and predominantly non-ischemic etiologies of pediatric heart failure may have contributed to the neutral findings. In another randomized clinical trial, Huang et al. evaluated the effects of carvedilol in children with heart failure and reported improvements in clinical symptoms, left ventricular ejection fraction, and cardiac prognostic biomarkers, but the results were not statistically significant [51].

Taken together, the available evidence suggests that beta-blockers may provide clinical benefit in selected pediatric patients. Current recommendations remain largely based on extrapolation from adult studies, highlighting the need for adequately powered pediatric trials to define the role of beta-blockers across the diverse etiologies of childhood heart failure.

The limited and often inconsistent efficacy of beta-blockers observed in pediatric heart failure has prompted investigators to explore the biological factors underlying these differences. Rossano and Shaddy highlighted several potential explanations, including age-dependent variations in drug pharmacokinetics and pharmacodynamics, developmental differences in myocardial biology, and the distinct pathophysiology and etiology of pediatric versus adult heart failure [35,61].

Developmental changes in drug disposition may substantially influence the therapeutic response to beta-blockers during childhood. Pharmacokinetic studies have reported that higher doses of carvedilol may be required in children to achieve systemic drug exposure and therapeutic effects comparable to that observed in adult patients, reflecting age-related differences in drug metabolism and clearance [1,50,62].

In addition, developmental differences in myocardial beta-adrenergic receptor expression and signaling have been proposed as another mechanism contributing to the response to beta-blocker therapy in children. Together, these findings suggest that the efficacy of beta-blockers cannot simply be extrapolated from adult populations and emphasize the need for pediatric-specific pharmacological studies to optimize treatment strategies for heart failure [1].

### 5.4. SGLT2 Inhibitors

In adult patients, several studies have evaluated the use of gliflozins with favorable outcomes. The landmark DAPA-HF trial showed that SGLT2 inhibitors reduced mortality and symptom burden, while improving quality of life. Similar findings were seen in the EMPEROR-Reduced trial. These findings supported the widespread use of SGLT2 inhibitors in adult patients with heart failure as a class I, Level A recommendation [3].

In pediatric patients, experience with SGLT2 inhibitors remains limited. Limited retrospective pediatric data suggest that SGLT2 inhibitors may be feasible and generally well tolerated in selected patients. Their efficacy and long-term safety remain uncertain in the absence of prospective randomized trials. Prospective randomized clinical trials are therefore needed to establish the efficacy and safety of SGLT2 inhibitors in pediatric patients [57].

### 5.5. Diuretics and Other Therapies

Diuretics remain one of the most frequently used drug classes in pediatric heart failure, particularly in patients whose clinical presentation is dominated by congestion [17,59]. Despite their widespread use in clinical practice, evidence supporting diuretic therapy in children with heart failure remains limited. Current recommendations are based on extrapolation from adult heart failure guidelines, complemented by clinical experience and expert consensus rather than pediatric-specific randomized clinical trials [17,19,37].

Ivabradine selectively inhibits the hyperpolarization-activated “funny” (If) current in sinoatrial node pacemaker cells, thereby reducing heart rate without directly affecting myocardial contractility [63]. Initially approved for the treatment of adult heart failure as a class IIa, Level B recommendation, ivabradine has subsequently been introduced into pediatric clinical practice, with growing evidence supporting its efficacy and safety in children with heart failure [22]. A pediatric randomized, double-blind, placebo-controlled trial demonstrated significant heart-rate reduction and reported improvements in clinical status, left ventricular ejection fraction, and quality-of-life measures [2]. These findings provide direct pediatric randomized evidence for potential adjunctive use of ivabradine in selected patients, although long-term pediatric evidence remains limited.

Nesiritide, a recombinant form of human B-type natriuretic peptide (BNP), has also been investigated as a potential therapeutic option for heart failure because of its vasodilatory, natriuretic, and diuretic properties [64]. However, despite its promising pharmacological profile, the clinical evidence supporting its use remains limited. The absence of adequately powered randomized controlled trials has resulted in continued uncertainty regarding its efficacy, and its routine use remains controversial even in adult patients [65].

Evidence in pediatric heart failure is even more limited. In a prospective study of children with decompensated heart failure, Jefferies et al. reported that nesiritide therapy was associated with improvements in renal function and reductions in cardiac biomarkers, suggesting potential clinical benefit [37]. Nevertheless, these findings should be interpreted with caution because of the limited number of available studies. Further randomized controlled trials are required before nesiritide can be considered part of routine pharmacological management of pediatric heart failure.

The evolution of heart failure pharmacotherapy has been markedly different in adults and children. In adult patients, therapeutic recommendations are supported by decades of randomized clinical trials and data, whereas in pediatric heart failure, the evidence remains considerably more limited. Consequently, the type and robustness of the available evidence differ substantially between adult and pediatric populations. Table 4 provides a comparative overview of the evidence supporting the major pharmacological therapies in adult and pediatric heart failure.

## 6. Challenges in Translating Adult Evidence to Pediatric Practice

### 6.1. Distinct Disease Biology

Pediatric heart failure should not be regarded simply as an earlier manifestation of adult disease. It represents a distinct biological entity requiring therapeutic approaches supported by pediatric-specific evidence rather than exclusive reliance on extrapolation from adult clinical trials.

These differences encompass disease etiology, myocardial development, molecular signaling pathways and cardiac remodeling [6,17,38,39].

### 6.2. Pharmacokinetics and Pharmacodynamics

Age-related differences in pharmacokinetics and pharmacodynamics represent other major challenges in translating adult heart failure therapies to pediatric patients. Drug absorption, distribution, metabolism and elimination undergo continuous maturation throughout childhood, resulting in substantial variability in systemic drug exposure and therapeutic response [53,61]. Children cannot be considered pharmacologically equivalent to adults and dosing strategies derived from adult populations may not achieve comparable efficacy and safety profiles [15].

Changes in hepatic enzyme activity, renal clearance and body composition influence the pharmacokinetics of many cardiovascular drugs. As a result, pediatric patients often require individualized dosing strategies that differ from adult practices [61].

Beta-blockers provide a good example of these challenges. Developmental variations in beta-adrenergic receptor density and signaling have been proposed as potential explanations for the inconsistent clinical results observed in pediatric therapies. Simpson et al. concluded in 2012 that the available evidence remains insufficient to support the routine use of beta-blockers in pediatric heart failure, emphasizing the need for pediatric-specific clinical trials [23]. Likewise, other studies have identified multiple factors limiting the translation of adult evidence, including differences in heart failure etiology, myocardial biology, drug metabolism and receptor signaling, conclusions that are consistent with earlier observations by Bruns [1,61].

Beta-blockers represent one of the clearest examples of developmental pharmacology. Pharmacokinetic studies have demonstrated that children require proportionally higher doses of carvedilol than adults to achieve comparable systemic drug exposure, reflecting age-dependent differences in drug metabolism and clearance [50,62]. Furthermore, developmental differences in myocardial beta-adrenergic receptor expression and signaling may further modify the pharmacodynamic response to beta-blockade. Together, these findings illustrate why therapeutic efficacy cannot be assumed to be equivalent across age groups and reinforce the need for further pharmacological studies in pediatric heart failure.

### 6.3. Challenges in Pediatric Clinical Trials

Several factors have been proposed to explain the technical and methodological challenges associated with conducting clinical trials in pediatric heart failure. Perhaps the greatest limitation is the relatively small number of children diagnosed with heart failure compared to the adult population. Although tertiary pediatric cardiology centers manage a substantial number of affected patients, the overall incidence of pediatric heart failure remains low, making patient recruitment particularly challenging [66,67]. Consequently, most pediatric studies are limited by small sample sizes, reducing the statistical power and limiting the ability to detect clinically meaningful treatment effects.

In addition to the rarity of the disease, the marked heterogeneity of pediatric heart failure further complicates clinical trial designs. Differences in the underlying etiology, developmental stage, myocardial biology and age-dependent pharmacokinetic and pharmacodynamic responses contribute to substantial variability in treatment outcomes [1,15]. These factors make it difficult to establish homogenous study populations and may partly explain why therapies that have demonstrated clear benefits in adults have yielded inconsistent results in pediatric clinical trials.

As a result, despite the widespread adoption of adult heart failure therapies in pediatric clinical practice, robust evidence supporting their efficacy and safety in children remains limited. Current treatment recommendations therefore continue to rely largely on extrapolation from adult studies, highlighting the urgent need for adequately powered, multicenter pediatric clinical trials specifically designed to address the unique characteristics of childhood heart failure.

Table 5 provides a comprehensive overview of the primary studies and secondary sources included in this review, together with their respective contributions to the evidence regarding epidemiology, etiology, diagnosis and management of heart failure.

## 7. Future Perspectives

Despite remarkable advances in adult heart failure therapy, the evidence supporting pharmacological management in children remains limited. Future progress will depend on the generation of high-quality pediatric-specific evidence rather than continued reliance on extrapolation from adult clinical trials [32,68].

Well-designed randomized controlled trials are essential to establish the efficacy, safety, and optimal dosing of contemporary heart failure therapies across the diverse spectrum of pediatric cardiovascular diseases. Because pediatric heart failure is a relatively rare condition, international multicenter collaborations will be crucial to overcome the limitations of small patient populations. Standardization of inclusion criteria, clinically relevant pediatric endpoints, and harmonized study protocols may facilitate recruitment and improve the quality and generalizability of future clinical trials [1,57,59,69,70].

Given the rarity and heterogeneity of pediatric heart failure, prospective registries and international collaborative research networks will play an increasingly important role in generating real-world evidence. Large multicenter databases can complement randomized clinical trials by providing valuable information regarding treatment effectiveness, long-term safety, and disease progression across diverse pediatric populations. These collaborative initiatives may accelerate evidence generation and facilitate the development of future evidence-based clinical guidelines [57,71].

Ultimately, the future of pediatric heart failure management will depend on replacing empirical treatment strategies with therapies supported by robust pediatric evidence. Integrating advances in developmental biology, precision medicine, and international collaborative research may provide an important opportunity to bridge the longstanding evidence gap between adult and pediatric heart failure and to improve outcomes for children affected by this complex syndrome [68].

## 8. Conclusions

Heart failure remains a major cause of morbidity and mortality across all age groups, yet important differences exist between adult and pediatric populations regarding disease etiology, pathophysiological processes and therapeutic response. Although contemporary pharmacological management targets similar neurohormonal pathways in both populations, the evidence supporting these therapies differs substantially. While adult heart failure treatment is supported by numerous large randomized clinical trials and well-established GDMT, pediatric recommendations continue to rely largely on extrapolation from adult studies, observational evidence and expert consensus.

The marked heterogeneity of pediatric heart failure, together with developmental differences in myocardial physiology, pharmacokinetics and pharmacodynamics, highlights the limitations of directly translating adult evidence into pediatric clinical practice. Bridging this evidence gap will require well-designed, multicenter pediatric clinical trials, international collaborative research networks and continued advances in developmental cardiovascular biology. Generating robust evidence is essential in optimizing pharmacological therapy and ultimately improving outcomes for children with heart failure.

## Figures and Tables

**Table 1:** Major etiologies of heart failure in adult and pediatric populations.

Adults	Children
Ischemic heart disease (coronary artery disease)	Congenital heart disease (left-to-right shunts, obstructive lesions, single ventricle physiology, complex CHD)
Hypertensive heart disease	Primary cardiomyopathies (genetic, familial, etc.)
Valvular heart disease	Secondary cardiomyopathies (metabolic, mitochondrial, neuromuscular, endocrine, etc.)
Arrhythmias and conduction disorders	Myocarditis
Cardiomyopathies (dilated, hypertrophic, others)	Genetic syndromes associated with cardiomyopathy
Endocrine and metabolic disorders (diabetes mellitus, thyroid disease)	Kawasaki disease
Chronic kidney disease	Systemic inflammatory and autoimmune diseases
Chronic pulmonary disease	Systemic infections
Infiltrative diseases (amyloidosis, sarcoidosis, hemochromatosis)	Pulmonary hypertension

**Table 2:** Key differences between adult and pediatric heart failure.

Characteristic	Adults	Children
Predominant etiology	Ischemic heart disease	Congenital heart disease
Etiological heterogeneity	Moderate	Very high
Disease type	Acquired	Genetic
Reversibility	Less frequent	More frequent

**Table 3:** Landmark pediatric randomized clinical trials.

Drug	Trial	Patients	Main Findings
Carvedilol	Shaddy, 2007 [50]	161	No statistically significant effect on primary clinical endpoint
	Huang, 2013 [51]	89	Positive clinical and paraclinical outcomes Not statistically significant
Ivabradine	Bonnet, 2017 [2]	116	Significant improvement in clinical status and cardiac function
ARNIs	PANORAMA-HF [52]	375	No superiority over enalapril

Abbreviations: ARNIs, angiotensin receptor and neprilysin inhibitors.

**Table 4:** Comparison of the evidence supporting pharmacological therapies in adult and pediatric heart failure.

Drug Class	Adults	Children	Interpretation for Pediatric Practice
ACE inhibitors	Standard of care Multiple landmark RCTs demonstrated reduced mortality, hospitalization and reverse remodeling	Supported by observational and small prospective studies	Current practice is supported by pediatric observational data, clinical experience, expert consensus, and extrapolation from adult evidence
ARBs	Alternative to ACE inhibitors Supported by large RCTs	Limited use	Pediatric use is largely extrapolated from adult evidence; pediatric efficacy and safety evidence base remains limited
ARNIs	Strong evidence RCTs (PANORAMA-HF)	PANORAMA-HF demonstrated clinical improvement but no superiority over enalapril	Superiority over conventional ACE-inhibitor therapy has not been established
Beta-blockers	Strong evidence from multiple RCTs (MERIT-HF, CIBIS-II, COPERNICUS)	Mixed results Largest RCT [50] showed neutral results	Uncertain pediatric efficacy; current recommendations also rely on observational evidence, expert consensus, and extrapolation from adult studies
MRAs	Strong evidence from multiple RCTs (RALES, EMPHASIS-HF)	Frequently used as adjunctive therapy	Frequently used as adjunctive therapy
SGLT2 inhibitors	Multiple positive RCTs (DAPA-HF, EMPEROR-Reduced trial)	Emerging experience	Preliminary data suggest feasibility and tolerability in selected patients, but pediatric efficacy and long-term safety remain uncertain
Ivabradine	Supported by SHIFT trial	RCT [2] demonstrated reduced heart rate and improved clinical status	One of the few therapies with direct pediatric randomized evidence; long-term and broader pediatric outcome data remain limited
Nesiritide	Controversial	Investigational Small pediatric studies suggest potential benefit	Evidence is insufficient to support routine use in pediatric HF

Abbreviations: ACE, angiotensin-converting enzyme; RCT, randomized clinical trial; ARBs, angiotensin receptor blockers; ARNIs, angiotensin receptor and neprilysin inhibitors; MRAs, mineralocorticoid receptor antagonists; SGLT2 inhibitors, sodium-glucose cotransporter-2 inhibitors.

**Table 5:** Overview of studies and secondary sources included in the review.

Author, Year	Publication Type	Population	Sample Size (*n*)	Topic	Main Findings	Role in Review
Rossano, 2014 [1]	Review	Children	N/A	Etiology and treatment	Further research is required to improve the outcomes of pediatric HF	Background
Bonnet, 2017 [2]	Randomized, double-blind, placebo-controlled study	Children	116	Treatment—Ivabradine	Ivabradine reduced heart rate and has a good safety profile	Efficacy/Safety evidence
McDonagh, 2021 [3]	Guideline	Adults	N/A	Acute and chronic heart failure management	Recommendations for each drug class	Background Identification of primary studies
Herath, 2015 [4]	Observational retrospective study	Children	35	Etiology and outcome	Familial DCM was associated with an unfavorable prognosis	Contextual evidence
Shaddy, 2018 [5]	Review	Children	N/A	The incidence and prevalence of heart failure	Standard definitions for heart failure are needed for future epidemiological studies	Contextual evidence
Price, 2019 [6]	Review Recommendation	Children	N/A	Etiology, diagnosis, treatment	Discussing treatment strategies depending on evidence level	Background Identification of primary studies
Kantor, 2013 [7]	Guidelines	Children	N/A	Heart failure management	Recommendations regarding treatment of pediatric heart failure	Background Identification of primary studies
Sorescu, 2025 [8]	Observational retrospective study	Children	68	Treatment—Intravenous immunoglobulin	Intravenous immunoglobulin improves clinical and paraclinical parameters in acute myocarditis	Efficacy/Safety evidence
Hänselmann, 2020 [9]	Review	Children	N/A	Etiology, diagnosis, treatment	Improving diagnosis can lead to more efficient management and favorable outcomes	Background
Lutokhina, 2025 [10]	Cohort study	Children	342	Prevalence and clinical implications of myocarditis in patients with cardiomyopathies	Myocarditis represents the most important epigenetic factor in the onset of genetic cardiomyopathies	Contextual evidence
Goldberg, 2016 [11]	Observational retrospective study	Children	172	The effect of anemia in acute heart failure	Anemia was prevalent in patients with acute heart failure and associated worse outcomes	Contextual evidence
Maiya, 2008 [12]	Observational retrospective study	Children	16	The effect of hypocalcemia and vitamin D deficiency in heart failure	Vitamin D deficiency associated worse outcomes	Contextual evidence
Sorescu, 2026 [13]	Case report	Children	1	Etiology and treatment	Etiology is one of the most important prognostic factors and determines treatment	Contextual evidence
Nakano, 2020 [15]	Review	Children	N/A	Treatment and outcomes of heart failure	HF represents a significant public health issue	Background
Bottner, 2018 [16]	Review	Children	N/A	Etiology, diagnosis, treatment	Treatment recommendations are based on extrapolated data from adult patients	Background
Watanabe, 2020 [17]	Review	Children	N/A	Etiology, diagnosis, treatment	Treatment recommendations are based on extrapolated data from adult patients	Background
Rusconi, 2010 [18]	Observational retrospective study	Children	24	Treatment—Carvedilol	Carvedilol improves cardiac function and symptoms	Efficacy/safety evidence
Masarone, 2017 [19]	Recommendation	Children	N/A	Diagnosis and management of heart failure	HF management requires optimization	Contextual evidence Identification of primary studies
Mille, 2023 [20]	Review	Children	N/A	Diagnosis and management of acute heart failure	Optimizing diagnosis and management improves outcomes	Background
Simpson, 2012 [23]	Review	Children	N/A	Relevance of adult studies in pediatric heart failure	Therapies for adults are not necessarily applicable to pediatric patients	Background Identification of primary studies
Silva, 2010 [24]	Review	Children	N/A	Treatment	Pediatric patients benefit from advances in the treatment of adult HF	Background Identification of primary studies
Rupp, 2018 [25]	Review	Children	N/A	Treatment	Certain drug classes are widely used in pediatric patients, despite not having sufficient evidence	Background Identification of primary studies
Weng, 2021 [26]	Meta-analysis of randomized controlled studies	Children Adults	N/A	Treatment—L-carnitine	L-carnitine is an effective adjunctive therapy in DCM	Efficacy evidence
Kocharian, 2009 [27]	Randomized, controlled, double-blind study	Children	38	Treatment—Coenzyme Q10	Administration of coenzyme Q10 improved cardiac function	Efficacy evidence
Soongswang, 2005 [28]	Prospective observational study	Children	15	Treatment—Coenzyme Q10	Administration of coenzyme Q10 can improve clinical status, but it did not improve cardiac function	Efficacy evidence
Recla, 2019 [30]	Review	Children	N/A	Treatment	Treating pediatric HF lacks evidence-based clinical trials.	Background
Kantor, 2010 [32]	Guideline	Children	N/A	Diagnosis and management	Specific treatment recommendations are given based on etiology	Contextual evidence
Snipelisky, 2019 [33]	Review	Adults	N/A	Diagnosis and management	HF management is heterogenous and depends on clinical severity, etiology and pathophysiology	Background
Hinton, 2017 [34]	Review	Children	N/A	Etiology, diagnosis and treatment	Additional studies are required to reach a consensus	Background Identification of primary studies
Rossano, 2014 [35]	Review	Children	N/A	Etiology and treatment	Ongoing research is imperative for improving outcomes in pediatric heart failure	Background Identification of primary studies
Jefferies, 2010 [37]	Review	Children	N/A	Diagnosis and treatment	Outcomes have not improved, despite treatment advancement	Background
Kemp, 2012 [38]	Review	Children	N/A	Pathophysiology	Further research to find targeted therapies can improve outcomes	Contextual evidence
Hsu, 2009 [39]	Review	Children	N/A	Diagnosis and treatment	Extrapolation from adult data may not be appropriate in HF	Background
Olsen, 2025 [40]	Review	Children	N/A	Treatment and outcome	Morbidity and mortality in pediatric heart failure remain higher than those of adults.	Background Identification of primary studies
Knudson, 2016 [42]	Review	Children	N/A	Pathophysiology	Understanding the pathophysiology of HF can lead to a better understanding of the management	Background Contextual evidence
Kadiyani, 2025 [44]	Review	Children	N/A	Treatment and outcome	Research prioritizing children is needed to improve outcomes	Background Identification of primary studies
Rogers, 2015 [43]	Review	Children	N/A	Pathophysiology, diagnosis and treatment	Pediatric HF constitutes an important public health issue	Background Identification of primary studies
Li, 2026 [45]	Review	Children	N/A	Pathophysiology, diagnosis and treatment	Further research should be based on standardization, validation and implementation	Background Identification of primary studies
Koubský, 2024 [46]	Review	Children	N/A	Diagnosis and treatment	Medical management is largely based on adult studies	Background Identification of primary studies
Bottner, 2018 [16]	Review	Children	N/A	Diagnosis and treatment	Management of pediatric HF should be multidisciplinary	Background
Schranz, 2024 [48]	Review	Children	N/A	Diagnosis and treatment	Medical management is largely based on adult studies	Background
Schultheiss, 2019 [49]	Review	Children	N/A	Pathophysiology, diagnosis, management	Determining etiology is necessary to improve medical management	Background
Momma, 2006 [53]	Review	Children	N/A	Treatment—ACE inhibitors	ACE inhibitors improve clinical status and cardiac function and have a good safety profile	Background Identification of primary studies
Rupp, 2014 [54]	Review	Children	N/A	Treatment	Pediatric HF management has advanced poorly compared to adult management	Background
Mori, 2000 [55]	Prospective controlled study	Children	24	Treatment—ACE inhibitors	ACE inhibitors are useful in improving echocardiographic parameters	Efficacy evidence
Patterson, 2017 [56]	Retrospective observational study	Adults	346	Treatment—MRAs	MRAs are underutilized in the treatment of heart failure	Contextual evidence
Shaddy, 2024 [52]	Randomized, controlled, double-blind trial	Children	375	Treatment—Sacubitril/Valsartan	The efficacy of sacubitril/valsartan did not exceed the efficacy of enalapril	Efficacy and safety evidence
Das, 2025 [57]	Review	Children	N/A	Treatment	There is an urgent need to individualize HF therapy	Background Identification of primary studies
Shaddy, 2007 [50]	Randomized controlled trial	Children	161	Treatment—Carvedilol	Carvedilol did not improve clinical status and outcomes in HF	Efficacy and safety evidence
Huang, 2013 [51]	Prospective, randomized, controlled study	Children	89	Treatment—Carvedilol	Carvedilol improved clinical status and cardiac function	Efficacy and safety evidence
Bruns, 2002 [61]	Review	Children	N/A	Treatment—beta-blockers	There are important differences in the PD and PK in children, compared to adults	Background Identification of primary studies
Albers, 2008 [62]	Prospective controlled study	Children	41	Treatment—Carvedilol	Carvedilol PK depends on age and weight	Contextual evidence
Cohen, 2020 [63]	Review	Children	N/A	Treatment	Interventional therapies can be used as a clinical bridge or an endpoint in management	Contextual evidence
Jefferies, 2007 [64]	Prospective observational study	Children	63	Treatment—Nesiritide	Nesiritide showed an acceptable safety profile and was associated with improvements in cardiac biomarkers	Efficacy and safety evidence
Moffett, 2006 [65]	Review	Children	N/A	Treatment	Pharmacogenomic studies are necessary on new and preexisting therapies for HF	Background
Neumann, 2009 [66]	Retrospective observational study	Children Adults	N/A	Epidemiology and public health	HF has increased incidence in the population	Contextual evidence
Schranz, 2020 [67]	Review	Children	N/A	Treatment	There is a current compromise between evidence-based and pathophysiology-based procedures	Background Contextual evidence
Ahmed, 2025 [68]	Review	Children	N/A	Treatment	Etiological heterogeneity and the rarity of the disease limit the development of effective treatment strategies	Background Contextual evidence
Everitt, 2014 [69]	Retrospective observational study	Children	868	Treatment and outcome	Etiology can influence treatment and outcome	Efficacy evidence
Rossano, 2020 [70]	Retrospective observational study	Children	557	Outcome and risk factors	Elevated heart rate can represent a risk factor	Contextual evidence

Abbreviations: ACE, angiotensin-converting enzyme; MRAs, mineralocorticoid receptor antagonists; HF, heart failure; DCM, dilated cardiomyopathy.

## Data Availability

No new data were created or analyzed in this study. Data sharing is not applicable to this article.
